# Supplementary material for: Robust determinants of income distribution across and within countries
Source: PLoS One. 2021 Jul 1;16(7):e0253291. doi: 10.1371/journal.pone.0253291 (PMC8248696; doi:10.1371/journal.pone.0253291)
Supplement: S1 File — (DOCX) [file pone.0253291.s002.docx]

Supplementary files include do files, appendix, data files, and readme file.

Do files:

- GiniKuz2020FE.do is the do file for transferring the panel data into quasi-cross-sectional and regressions by fixed-effect time form.
- GiniKuz2020TREND.do is the do file for transferring the panel data into quasi-cross-sectional and regressions by trended time form.
- Replication.do is the do file for literature replication.

M file, simulation.m is the m file for simulation of the total marginal effects of capital terms.

Data:

- GiniKuzAvgData2020.dta includes the panel data of all variables to run regressions.
- RawData is the excel file including all data sources, sheet name is the data source.

Appendix: the excel file including all tables of data summary and regression details. File sheets are named by the table caption.
